# Integrating digital risk communication and indigenous knowledge in megathrust mitigation in Sukabumi Regency

**DOI:** 10.4102/jamba.v18i1.2149

**Published:** 2026-09-18

**Authors:** Deddy Irwandy, Fajar I. Mirza, Yoseph W. Kurniawan, Camila A. Komar, Anindya A.C. Nugraha

**Affiliations:** 1 Department of Communication, Faculty of Communication, LSPR Institute of Communication and Business, Jakarta, Indonesia

**Keywords:** megathrust risk, people-centred early warning system, participatory risk communication, indigenous knowledge, social capital

## Abstract

Indonesia’s southern Java coast faces a substantial threat from megathrust earthquakes and near-field tsunamis, where warning times are limited, and community preparedness remains uneven. This study examines how integrating digital communication tools with local and indigenous knowledge can strengthen disaster risk communication and enhance preparedness in high-risk coastal communities. Using a convergent mixed-methods design with a participatory orientation, fieldwork was conducted in Sukatani and Karangpapak Villages, Sukabumi Regency, West Java, between July 2024 and August 2024. Data were collected through focus group discussions, semi-structured interviews with village officials, Regional Disaster Management Agency (BPBD) officers, traditional and community leaders, participatory observations, documentary analysis, and structured pre- and post-intervention questionnaires. The findings reveal three main patterns. Firstly, community understanding of megathrust risk was initially limited and influenced by fragmented information, diverse media framing and uneven disaster literacy. Secondly, village officials and traditional leaders served as trusted intermediaries by translating technical early warning information and locally embedded environmental knowledge into socially meaningful messages. Thirdly, participatory communication interventions were associated with improvements in disaster knowledge, preparedness and community-led initiatives, including WhatsApp-based coordination and locally organised evacuation procedures.

**Contribution:**

These findings suggest that effective disaster risk communication depends not only on technological capacity but also on social trust, local leadership and culturally grounded communication practices that strengthen collective preparedness and community resilience.

## Introduction

Indonesia is widely recognised as one of the countries with the highest levels of natural hazard vulnerability in the world because of its complex geological setting and extensive coastal exposure. The southern coastal region of Java Island faces a significant threat from megathrust earthquakes that may trigger large-scale tsunamis. Studies conducted by the Meteorology, Climatology and Geophysics Agency (BMKG) and the Indonesian Institute of Sciences (LIPI) indicate that the potential magnitude of a megathrust earthquake along the Java subduction zone could reach 8.7–9.0 magnitude. Despite this high level of hazard exposure, community preparedness remains relatively low, particularly in rural coastal areas where access to disaster information and disaster literacy is limited. One region that exemplifies this condition is the Sukabumi Regency in West Java. The southern coastal area of Sukabumi is highly vulnerable to megathrust earthquakes and tsunami hazards. Its geographical position, directly adjacent to the Indian Ocean, places Sukabumi in an active subduction zone formed by the convergence of the Indo-Australian and Eurasian tectonic plates. Continuous tectonic pressure accumulation in this zone creates the potential for sudden and devastating releases of seismic energy. Furthermore, the presence of the active Cimandiri Fault, which crosses parts of the Sukabumi area, increases the likelihood of inland earthquakes that may compound the disaster impacts in densely populated settlements (BMKG 2023). Recent scientific assessments suggest that a megathrust earthquake in southern West Java could generate tsunami waves exceeding 20 m in height in several coastal sub-districts, including Cisolok, Pelabuhanratu, Ciemas, Surade and Tegalbuleud. In a worst-case scenario, tsunami waves could reach coastal land areas in less than 20 min following a major earthquake. Such conditions demand a high level of preparedness from both communities and local governments, particularly through disaster education, the provision of evacuation infrastructure and strengthening early warning systems (EWSs) (BPBD Kabupaten Sukabumi 2024; Radar Mojokerto 2025). Previous studies on near-field tsunami risk in Indonesia emphasise that EWSs are most effective when they adopt a people-centred approach that integrates community participation, local capacity and institutional coordination, particularly in coastal areas with limited warning times (Rahayu et al. 2020). Sukabumi thus represents a critical case for understanding how geological risk knowledge can be translated into effective disaster risk reduction practices to minimise casualties and material losses. Research on tsunami-prone communities in Indonesia indicates that limited initial knowledge and uneven disaster literacy remain persistent challenges, reinforcing the need for continuous risk communication and community-based educational initiatives (Aksa, Ashar & Siswanto 2024). Risk communication plays a crucial role in bridging scientific knowledge and community action within disaster risk reduction efforts by transforming hazard information into preparedness and protective behaviours. The effectiveness of EWSs depends not only on the availability of hazard information but also on how risks are communicated, interpreted and acted upon by at-risk communities (Fakhruddin et al. 2020). However, previous studies indicate that top-down risk communication approaches are often ineffective in fostering public understanding and meaningful community participation (Ali et al. 2021; Paton & Johnston 2001). A key challenge lies in the disconnect between technology-driven EWSs and the social and cultural realities of communities, where local values and indigenous knowledge systems continue to shape everyday risk perceptions and responses. The growing use of digital technologies, such as the InaRISK application, community-based WhatsApp groups and village radio networks, has demonstrated the potential to accelerate the dissemination of disaster-related information (Tompkins et al. 2023). Nevertheless, significant challenges persist, including unequal digital literacy, limited Internet connectivity and resistance to new technologies among older adults and other vulnerable groups. Simultaneously, indigenous knowledge systems that have been transmitted across generations, such as interpreting animal behaviour, wind patterns or environmental signs, remain trusted indicators of impending hazards within local communities (Mwalwimba, Manda & Ngongondo 2024). Despite their continued relevance, such knowledge systems are often marginalised or excluded from formal early warning mechanisms owing to perceptions of being unscientific. Research on tsunami risk reduction has further demonstrated that indigenous knowledge systems, including the recognition of sudden sea withdrawal, unusual wave conditions, changes in animal behaviour and locally transmitted knowledge of evacuation routes to higher ground, continue to play an important role in community preparedness. In many coastal communities, these environmental cues are interpreted collectively by traditional leaders and local residents to support early decision-making during potential tsunami events and should be recognised as complementary to scientific early warning mechanisms (Mikulecký et al. 2023). Community-Based Disaster Risk Reduction (CBDRR) offers a framework capable of bridging the gap between formal technological systems and locally grounded knowledge. Community-Based Disaster Risk Reduction emphasises community involvement in risk mapping, emergency planning, implementation and evaluation processes, prioritising participatory communication, social trust and the integration of scientific and indigenous knowledge (Shaw 2016). Studies on disaster risk reduction in Indonesia highlight the strategic role of local leadership and community actors in strengthening preparedness, coordination and trust, particularly in decentralised governance contexts (Ruslanjari et al. 2024). Within people-centred EWS frameworks, communities are positioned not only as recipients of warnings but also as active producers and distributors of risk knowledge. Empirical evidence from coastal communities in other disaster-prone regions similarly shows that community-based EWSs enhance preparedness when local actors are actively involved in warning dissemination, interpretation and response planning (Pham, Thieken & Bubeck 2024). However, evidence suggests that EWS initiatives frequently struggle with sustainability when social contexts, such as local power relations, social capital and informal communication networks, are insufficiently considered (Nyahunda 2024). Against this backdrop, this study examines how the integration of digital communication tools and local knowledge systems can strengthen EWSs and enhance community preparedness in high-risk coastal areas, drawing on empirical evidence from Sukatani and Karangpapak Villages in Sukabumi Regency. By advancing a participatory risk communication approach grounded in socio-cultural realities, this study contributes to disaster risk studies by demonstrating pathways for reducing vulnerability and strengthening community resilience in megathrust-prone regions. Within this context, risk communication is not merely a supporting activity but a core component of disaster risk reduction and resilience building, as it shapes how hazard information is interpreted, trusted and translated into preparedness and collective action at the community level.

## Research methods and design

### Research design and study area

This study employed a convergent mixed-methods design, integrating qualitative and quantitative approaches to examine how digital risk communication and indigenous knowledge contribute to community preparedness in megathrust-prone coastal communities (Creswell & Creswell 2018). The qualitative component served as the primary approach, focusing on exploring participants’ experiences, communication practices and locally embedded knowledge through focus group discussions (FGDs), in-depth interviews, participatory observations and documentary analysis. The quantitative component complemented these findings through pre- and post-intervention questionnaires, enabling the assessment of changes in community knowledge and preparedness following participatory risk communication activities. Both datasets were collected concurrently during the July–August 2024 fieldwork period and integrated during the interpretation stage to provide a comprehensive understanding of disaster risk communication.

The study adopted a participatory orientation that actively engaged community members, village officials and local stakeholders throughout the research process. This approach enabled the researchers not only to document how disaster risk information was communicated but also to understand how it was interpreted, negotiated and translated into collective preparedness within the local socio-cultural context. By combining participatory engagement with multiple sources of evidence, the study captured both the social processes underlying community preparedness and measurable changes in disaster-related knowledge following the intervention.

The research was conducted in two coastal villages in Sukabumi Regency, West Java: Sukatani Village in Surade District and Karangpapak Village in Cisolok District. Both villages are located along the southern coast of Java and face the Indian Ocean, placing them in close proximity to the Sunda megathrust zone, one of Indonesia’s most seismically active subduction systems. These locations were purposively selected because of their high exposure to megathrust earthquake and tsunami hazards, as well as their strong socio-cultural characteristics, including the continued transmission of indigenous knowledge related to environmental hazard recognition and community-based evacuation practices. Both villages represent typical rural coastal settings where formal disaster management systems intersect with local leadership structures, indigenous knowledge and community-based communication networks, making them appropriate sites for examining the integration of digital and locally grounded disaster risk communication.

### Participants and data collection

This study involved multiple participant groups representing both community members and institutional stakeholders to obtain comprehensive insights into community-based disaster risk communication. A total of 48 community members participated in the FGDs, organised into six discussion groups consisting of eight participants each, representing diverse age groups (18–65 years), occupations and gender. In addition, semi-structured interviews were conducted with 16 purposively selected key informants, including village heads (*n* = 2), village secretaries (*n* = 2), BPBD officers (*n* = 3), traditional leaders (*n* = 4), Rukun Tetangga (RT) and Rukun Warga (RW), (the neighbourhood-level administrative units in Indonesia) leaders (*n* = 3) and NGO representatives (*n* = 2), allowing both institutional and community perspectives to be explored.

Data were collected between July 2024 and August 2024 using complementary qualitative techniques, including FGDs, in-depth interviews, participatory observations, documentary analysis and participatory risk communication activities. The FGDs explored participants’ knowledge, perceptions and communication practices regarding megathrust and tsunami risks, whereas the interviews examined how disaster information was communicated through formal institutions and informal community networks. Participatory observations were undertaken during disaster awareness sessions and evacuation simulations, while documentary analysis focused on communication materials, visual media, meeting records and disaster communication guidelines produced by BPBD and partner institutions.

The quantitative component consisted of structured pre- and post-intervention questionnaires administered to 120 respondents, with equal representation from Sukatani Village (*n* = 60) and Karangpapak Village (*n* = 60). Respondents were selected using stratified random sampling to ensure representation across the hamlets. The questionnaire was developed specifically for this study by the research team to measure community knowledge of megathrust risk, understanding of EWSs and perceptions of disaster communication channels. Pre-intervention questionnaires were completed by all respondents, whereas post-intervention questionnaires were completed by 108 participants, yielding a response rate of 90%. The intervention activities enabled changes in disaster knowledge and preparedness to be assessed within the same participant group.

### Data analysis

Qualitative data from the FGDs, interviews, observations and documentary materials were analysed using thematic analysis following the six-phase framework proposed by Braun and Clarke (2006). All interview and FGD recordings were transcribed verbatim in Indonesian and translated into English where necessary. The researchers repeatedly reviewed the transcripts to familiarise themselves with the dataset before conducting the coding process using NVivo 12 (QSR International Pty Ltd, Melbourne, Australia) software to identify recurring patterns relevant to disaster risk communication and community preparedness.

During the coding process, 87 initial codes were generated, representing recurring concepts related to trust in traditional leaders, digital communication practices, indigenous knowledge of environmental hazard recognition, disaster literacy and community preparedness. These codes were subsequently organised into 12 candidate themes, which were reviewed through iterative discussions among the research team to evaluate conceptual overlap, internal consistency and empirical relevance across the entire dataset.

Following the refinement process, overlapping themes were merged, and themes with limited empirical support were removed, resulting in three final analytical themes that formed the basis of the Results section: (1) limited initial community knowledge of megathrust risk, (2) the strategic role of village officials and traditional leaders in risk communication and (3) increased knowledge and preparedness following communication interventions. Intercoder reliability was assessed through collaborative coding, achieving a Cohen’s kappa coefficient of 0.82, indicating substantial agreement among the researchers. Quantitative questionnaire data were analysed using descriptive statistics to summarise respondent characteristics and compare levels of disaster knowledge before and after communication intervention. The quantitative findings were used to complement the qualitative findings by describing changes in community knowledge and preparedness following the participatory communication activities. Qualitative and quantitative findings were subsequently integrated during the interpretation stage to provide a comprehensive understanding of how participatory risk communication influenced community preparedness in the two study villages.

### Trustworthiness and research rigour

The trustworthiness of the qualitative findings was strengthened through triangulation of data sources and methods by comparing evidence obtained from FGDs, in-depth interviews, participatory observations, documentary analysis and questionnaire findings. The use of multiple data sources enabled the researchers to examine the consistency of emerging patterns across different participants and forms of evidence, thereby enhancing the credibility of the findings and reducing the influence of single-source interpretations.

To strengthen analytical rigour, member checking was conducted with selected key informants to confirm the accuracy of the researchers’ interpretations and ensure that the findings reflected participants’ experiences. Peer debriefing was also undertaken among the research team throughout the coding and theme refinement process to discuss analytical decisions, resolve coding discrepancies and improve the consistency of thematic interpretation. These procedures enhanced the dependability and confirmability of the qualitative analysis.

An audit trail documenting major methodological and analytical decisions was maintained throughout the research process to ensure transparency and allow the analytical procedures to be traced systematically. The integration of qualitative and quantitative findings further strengthened the overall rigour of the study by providing complementary evidence on community preparedness and disaster risk communication, thereby supporting a more comprehensive interpretation of the research findings within the study context.

### Ethical considerations

The study complied with ethical standards for social research involving human participants and received ethical exemption approval from the Research Ethics Committee (Komite Etik Penelitian – KEP), Institute for Research and Community Service (LPPM), Universitas Negeri Malang, under ethical clearance number 9.02.17/UN32.14.2.8/LT/2026. The protocol was declared ethically appropriate in accordance with the World Health Organization (2011) ethical standards and the Council for International Organizations of Medical Sciences (CIOMS) (2016) guidelines, which include considerations of social value, scientific validity, equitable risk–benefit assessment, protection against exploitation, confidentiality and privacy protection, and informed consent. All participants were informed about the purpose of the research and voluntarily agreed to participate in interviews, FGDs and questionnaire surveys. Anonymity and confidentiality were maintained throughout the research process, and all data were reported in aggregated form to prevent the identification of individuals.

## Results

The findings of this study suggest that integrating digital communication tools and local knowledge within disaster risk reduction practices in Sukatani and Karangpapak Villages illustrates an adaptive risk communication approach that responds to local needs and socio-cultural contexts. The results indicate that preparedness and risk awareness are shaped not only by access to information technologies but also by how disaster knowledge is framed, trusted and communicated through locally embedded social networks. Three main findings emerged from this analysis.

### Limited initial community knowledge of megathrust risk

The initial FGDs revealed that most residents had a limited understanding of the concept of a megathrust earthquake as a tangible and imminent disaster threat. Large earthquakes and tsunamis were commonly perceived as events that ‘rarely occur’ and as risks beyond the scope of everyday community experiences. Consequently, disaster risk is often viewed as abstract rather than immediate. Information related to megathrust hazards circulated in fragmented forms, primarily through social media platforms and informal news sources, contributing to uneven and sometimes inconsistent awareness levels. These conditions indicate that while some level of risk recognition existed, it was not accompanied by a comprehensive or actionable understanding of the megathrust hazards. Media information significantly shapes community members’ early awareness and initial knowledge of megathrust risk. However, the narratives encountered by residents varied considerably depending on the framing adopted by the different media outlets. Analysis of selected media sources showed substantial variation in emphasis, tone and messaging related to megathrust hazards over time. Some media narratives focused on preparedness and mitigation, while others highlighted the scale and inevitability of potential disasters. This diversity in media framing has different implications for public understanding, ranging from heightened vigilance to increased anxiety or fatalistic attitudes, as illustrated in Table 1.

**TABLE 1:** Media framing analysis of megathrust risk information.

No	Media or News title	Main framing	Focus and keywords
1	‘Measuring Sukabumi’s preparedness facing potential megathrust and tsunami’ (Detik.com)	Preparedness and mitigation	Focus on preparedness: ‘when cannot be predicted’, ‘EWS’, ‘socialization’, ‘60 villages’, ‘30 schools’
2	‘BRIN researchers reveal the potential for earthquakes in 15 megathrust segments in Indonesia’ (BRIN via ‘Radar Sukabumi’)	Threat and large scale	Keywords such as ‘magnitude up to 9’, ‘15 segments’, ‘will occur’, ‘recurring cycle’
3	‘Megathrust earthquake looms over Indonesia, community … not ready to face the threat’ (YouTube news)	Public unpreparedness	‘looms’, ‘community not ready’, ‘will occur’

*Source:* Alamsyah, S., 2024, ‘Menakar Kesiapsiagaan Sukabumi Hadapi Potensi Megathrust dan Tsunami’ [Assessing Sukabumi’s Preparedness to Face Potential Megathrust and Tsunami], *detikJabar*, 15 September, viewed 16 September 2024, from https://www.detik.com/jabar/berita/d-7541321/menakar-kesiapsiagaan-sukabumi-hadapi-potensi-megathrust-dan-tsunami; Kompascom Reporter on Location, 2024, *Gempa Megathrust Membayangi Indonesia, Masyarakat Belum Siap Hadapi Bencana?* [*Megathrust earthquake looms over Indonesia, community not ready to face the threat?*], YouTube, viewed 25 August 2024, from https://www.youtube.com/watch?v=7xo9hAldX5o; Salam, H., 2024, ‘Peneliti BRIN Ungkap Potensi Gempa 15 Segmen Megathrust di Indonesia’ [BRIN Researchers Reveal Potential Earthquakes in 15 Megathrust Segments in Indonesia], *Radar Sukabumi*, 03 September [Online news article], viewed 05 September 2024, from https://radarsukabumi.com/nasional/peneliti-brin-ungkap-potensi-gempa-15-segmen-megathrust-di-indonesia/

EWS, early warning system; No., number.

This table shows the implications of diverse media framing for public awareness and understanding of megathrust risk. National media coverage, such as reports published by detik.com, tends to frame megathrust risk within a preparedness and mitigation narrative, encouraging vigilance and proactive preparation without generating excessive panic. In contrast, local media narratives, such as those disseminated through Radar Sukabumi, emphasise the magnitude and scale of potential megathrust events, creating the impression of a severe and imminent threat that may simultaneously trigger fear and heighten the urgency of policy and mitigation responses. Social media content, including YouTube-based news, frequently highlights community unpreparedness and the inevitability of disasters, which can stimulate awareness and action but may also contribute to increased public anxiety. Beyond differences in media framing, the qualitative findings revealed that community members interpreted disaster risk through locally embedded knowledge that had been transmitted across generations. Despite their limited familiarity with the scientific concept of megathrust earthquakes, discussions with community members revealed that many residents possessed locally transmitted knowledge for recognising potential tsunami hazards. Rather than relying solely on technical terminology, participants described environmental cues such as sudden sea withdrawal, unusual wave conditions, changes in animal behaviour and inherited knowledge of evacuation routes as practical indicators requiring immediate response. These findings suggest that indigenous knowledge remained embedded in everyday community experience and continued to shape initial interpretations of disaster risk before formal warnings were received:

‘We did not really understand the term “megathrust,” but our elders have always reminded us that if the sea suddenly recedes, the sound of the waves changes, or animals behave unusually, everyone should immediately move to higher ground because those are signs that danger may be approaching.’ (FGD participant, 52 years old, Sukatani Village)

The interview findings indicate that local knowledge functioned as an interpretive framework rather than a substitute for scientific information. Community members understood environmental signals through shared experience and collective memory, while official disaster information was regarded as necessary to confirm the level of risk and determine appropriate response measures. This interaction illustrates that indigenous knowledge and formal EWSs operated in complementary ways within local preparedness practices:

‘Natural signs are still important to us because they have been passed down through generations. However, when BPBD or the village office provides information, traditional leaders usually explain it in language that everyone understands, making people more confident about what actions should be taken.’ (Traditional leader, 58 years old, Karangpapak Village)

The integration of indigenous knowledge with trusted local communication channels explains why respondents consistently expressed greater confidence in village officials and traditional leaders than in digital platforms alone. Preparedness was therefore influenced not only by access to disaster information but also by the credibility of those interpreting and communicating risk within the community. These findings suggest that strengthening disaster literacy in coastal communities requires recognising local knowledge as a complementary resource that reinforces, rather than replaces, formal disaster risk communication and EWSs.

### Strategic role of village officials and traditional leaders in risk communication

Findings from the interviews indicate that village officials and traditional leaders performed complementary roles in communicating disaster risk within the study communities. Village officials acted as formal communication channels responsible for disseminating information from BPBD and the InaRISK platform, whereas traditional leaders contributed cultural legitimacy by interpreting technical messages through locally familiar language, values and community norms. This complementary relationship strengthened public confidence in disaster information and facilitated broader community engagement in preparedness activities:

‘People usually wait for explanations from the village office or traditional leaders before making decisions. Information from BPBD is important, but residents understand it more easily when it is explained using familiar language and connected to our local experience.’ (Village official, 50 years old, Sukatani Village)

The interview evidence demonstrates that the effectiveness of disaster communication depended not only on the availability of official information but also on the credibility of those delivering it. Scientific warnings acquired greater legitimacy when interpreted by trusted local actors who were able to connect technical explanations with community experience and indigenous knowledge. This finding highlights that social trust functioned as a critical mechanism linking formal EWSs with collective preparedness at the local level.

Despite their strategic position, village officials and neighbourhood leaders acknowledged limitations in their understanding of technology-based EWSs before the implementation of the participatory communication activities. Limited disaster literacy, insufficient familiarity with digital platforms such as InaRISK, and uneven communication skills reduced their capacity to translate official warnings into clear and actionable community messages. These findings indicate that strengthening local communication capacity remains essential for improving the effectiveness of people-centred EWSs in coastal communities.

### Increased knowledge and preparedness after communication intervention

Participatory risk communication activities were associated with improvements in community knowledge and preparedness following FGDs, disaster awareness sessions and evacuation simulations. Questionnaire findings indicated that a larger proportion of participants understood the concept of megathrust risk and the function of EWSs after the intervention than before the activities. Beyond individual learning, participants demonstrated greater confidence in discussing disaster risks and identifying appropriate preparedness measures within their communities:

‘During the simulations, we observed that residents became more confident in recognising evacuation procedures and responding to official warnings. They no longer viewed disaster information as something distant but began discussing preparedness as part of everyday community responsibility.’ (BPBD officer, 55 years old, Sukabumi Regency)

The qualitative findings suggest that the intervention contributed to changes extending beyond knowledge acquisition. Repeated discussions, simulations and community engagement helped participants connect scientific explanations with practical experiences, enabling them to interpret disaster information more critically and respond more confidently. This suggests that participatory communication encouraged learning through interaction rather than through one-way dissemination of information, thereby strengthening community readiness to face potential megathrust and tsunami hazards.

Improved preparedness was also reflected in the emergence of collective initiatives that strengthened communication and coordination within the villages. Participants described the establishment of WhatsApp-based communication groups, collaborative discussions on evacuation routes, and closer coordination between residents, neighbourhood leaders and village authorities during preparedness activities. These developments suggest that the communication interventions contributed to greater individual awareness while reinforcing collective action through locally organised communication networks:

‘After participating in the programme, residents became more active in sharing disaster information with neighbours. We agreed on evacuation routes together and used our community WhatsApp group to remind one another about preparedness activities, something that rarely happened before.’ (RT leader, 55 years old, Karangpapak Village)

These findings suggest that participatory communication interventions contributed to both cognitive and collective outcomes within the study communities. Enhanced understanding of disaster risks was accompanied by stronger collaboration, increased trust among local actors and more organised community coordination. Rather than functioning solely as educational activities, the interventions strengthened existing social networks and encouraged communities to integrate formal disaster information with locally embedded communication practices, thereby supporting the development of a more sustainable culture of preparedness in megathrust-prone coastal areas.

## Discussion

The findings of this study suggest that a hybrid risk communication approach combining digital technologies with local and indigenous knowledge can strengthen disaster preparedness in rural communities characterised by strong socio-cultural structures. Although this study is situated in coastal Indonesia, the findings are highly relevant to disaster-prone communities across the Global South, where technological EWSs often coexist with strong local knowledge systems, uneven institutional capacity and deeply embedded social trust networks. As observed in Sukabumi, integrating these knowledge systems facilitated the dissemination of disaster information and encouraged greater community engagement in local risk mitigation processes. This hybrid approach enabled communities to engage with scientifically grounded information while retaining culturally meaningful modes of interpretation, thereby supporting trust in locally communicated disaster information. Similar findings in disaster risk studies indicate that indigenous and local knowledge systems continue to play a crucial role in shaping preparedness and risk interpretation, particularly in tsunami-prone contexts where formal warning systems alone are insufficient to ensure community response (Aksa et al. 2024; Mikulecký et al. 2023). These findings align with existing research emphasising the value of indigenous knowledge in disaster risk reduction contexts (Mwalwimba et al. 2024). This study further supports the people-centred EWS framework, which positions communities as active participants rather than passive recipients of disaster information (Hermans et al. 2022). By involving community members in communication processes, EWSs become more responsive to local realities and social dynamics of the community. Empirical evidence from people-centred EWS research in Indonesia similarly demonstrates that community engagement and institutional coordination are decisive factors in enhancing the effectiveness of early warnings, particularly in near-field tsunami settings with limited response times (Rahayu et al. 2020). The participatory orientation observed in Sukabumi reflects a socially inclusive risk communication approach in which communities act as co-producers of risk knowledge, consistent with earlier studies highlighting the limitations of top-down communication models in disaster contexts (Paton & Johnston 2001). Recent disaster risk research further confirms that risk communication is a critical component of disaster risk reduction, as preparedness outcomes depend not only on the accuracy of hazard information but also on how risks are communicated, interpreted and trusted by at-risk communities (Fakhruddin et al. 2020). The findings suggest that the effectiveness of risk communication depends not only on technological capability but also on the capacity of communication systems to operate within the social realities and everyday communication practices of local communities. Digital platforms are likely to be less effective when they do not resonate with the community’s norms, values and everyday communication practices. This underscores the importance of integrating local leaders, community facilitators and accessible media channels into the design of risk communication strategies. Studies on community-based EWSs show that preparedness is significantly strengthened when local actors are directly involved in interpreting warnings, disseminating information and coordinating responses at the community level (Pham et al. 2024). Such an approach resonates with the communication-for-social-change model, which emphasises horizontal dialogue, shared reflection and collective learning as the foundations for building resilience. In the Sukabumi context, local leaders and community facilitators play critical roles in translating technical risk information into socially meaningful messages. Their involvement helps translate abstract disaster warnings into locally actionable knowledge. By embedding risk messages within familiar narratives and symbols, these actors enhance the credibility and clarity of the early warning information. These findings highlight that risk communication should be understood as both a social and technical process, particularly in rural communities exposed to disaster risk. This finding aligns with research from Indonesia, demonstrating that leadership by local actors and community figures enhances coordination, trust and collective action in disaster risk reduction processes, especially within decentralised governance contexts (Ruslanjari et al. 2024).

Despite its contributions, this study has some limitations that should be acknowledged. The research was conducted in a limited number of villages with a relatively small group of informants, which requires caution in generalising the findings to other contexts. Similar challenges related to uneven disaster knowledge and preparedness levels have been identified in other tsunami-prone communities, suggesting that such limitations reflect broader structural issues in coastal disaster risk contexts rather than isolated local conditions (Aksa et al. 2024). Moreover, risk communication dynamics are inherently contextual and may evolve in response to changes in local sociopolitical conditions. Nevertheless, the findings provide a valuable empirical basis for developing more structured and context-sensitive community-based risk communication strategies for disaster-prone regions of Indonesia. Although the findings are context-specific, they provide analytically transferable insights for other coastal and rural communities facing similar challenges related to near-field tsunami risk, social vulnerability and the integration of formal EWSs with local knowledge. The study also suggests that the integration of digital systems and local knowledge is influenced by existing levels of social capital and trust among community actors. Empirical studies in disaster-prone rural settings show that social capital plays a decisive role in building community resilience by strengthening trust, coordination and collective efficacy in preparedness and response activities (Xiong & Li 2024; Yi et al. 2024). Findings from this study indicate that community members were more responsive to disaster information communicated by socially trusted local actors, such as village officials and traditional leaders, than to information received exclusively through digital platforms. This pattern suggests that trust plays an important role in shaping how disaster-risk messages are received, interpreted and acted upon within local communities. The emphasis on trust underscores the importance of the human interface in technology-based risk communication systems. While digital applications such as InaRISK facilitate the dissemination of disaster information, their effectiveness in practice depends on the capacity of local actors to communicate risk information in ways that are contextually relevant and socially trusted. Evidence from Indonesia further indicates that higher levels of social capital and structured participation in disaster meetings significantly improve community responsiveness to early warning alerts, reinforcing the importance of participatory mechanisms in EWSs (Nugraha & Dewanti 2025). This finding suggests that EWSs should not be evaluated solely on their technological performance but also on their ability to integrate human relationships and social communication norms at the local level.

Strengthening local capacity, recognising indigenous knowledge and supporting appropriate digital technologies require sustained cross-sector collaboration. Recent studies have highlighted that social media and digital data can support disaster risk management by enhancing community engagement, situational awareness and feedback mechanisms, provided these tools are embedded within trusted local communication structures (Erokhin & Komendantova 2024). Disaster risk reduction policies should prioritise capacity-building initiatives that empower local actors to function as effective communicators and facilitators of disaster preparedness. Without such investment, technology-driven interventions risk reproducing existing information gaps and exclusions rather than contributing to meaningful reductions in vulnerability. Ultimately, the sustainability of risk communication strategies is closely linked to the ability of disaster management systems to foster trust-based communication across multiple societal levels. As emphasised by Paton and Johnston (2001), social trust is a decisive factor in shaping the acceptance and uptake of risk messages, particularly in communities with limited prior disaster experience. In the Sukabumi context, the development of trust-based communication networks involving BPBD, village officials and community leaders was crucial in transforming risk information into concrete preparedness actions. These findings suggest that disaster communication policies should explicitly integrate trust and social proximity as key considerations in the design and implementation of EWSs.

## Conclusion

This study set out to examine how the integration of digital communication tools and local knowledge systems can strengthen disaster risk communication and early warning effectiveness in megathrust-prone coastal areas of Indonesia. Drawing on a convergent mixed-methods design with a participatory orientation in Sukatani and Karangpapak Villages, the findings confirm that despite extremely high geological risk exposure, community preparedness remains constrained by limited initial knowledge, uneven disaster literacy and fragmented information flows. Media framing further shapes early risk perceptions in complex ways, alternately encouraging preparedness or amplifying fear, thereby underscoring that access to information alone is insufficient without trusted interpretation and contextualisation at the local level. The results suggest that risk communication in megathrust and near-field tsunami contexts is fundamentally a socio-cultural process rather than a purely technological one. Village officials and traditional leaders emerged as critical intermediaries linking formal EWSs, such as BPBD and InaRISK, with community understanding and action. Their moral authority, social legitimacy and proximity to residents enabled the translation of scientific risk information into locally meaningful messages. The findings also identified capacity gaps among these actors, particularly regarding digital early warning technologies, highlighting the importance of sustained training and institutional support to strengthen their ability to communicate disaster information accurately, contextually and in ways that encourage collective preparedness. Participatory communication interventions, including FGDs, training sessions and community simulations, were associated with improvements in disaster knowledge, preparedness and collective capacity. The observed increase in post-intervention understanding, together with the emergence of community-led communication networks and locally coordinated evacuation initiatives, suggests that participatory risk communication can encourage communities to move beyond passive recipients of disaster warnings towards active contributors to community-based preparedness. Beyond individual learning outcomes, these processes strengthened social capital, reinforced coordination among local actors and contributed to the development of a more adaptive preparedness culture embedded within everyday community interactions. Overall, this study contributes to disaster risk studies by showing that effective EWSs in megathrust-prone regions can be strengthened through hybrid socio-technical approaches that integrate digital communication technologies with indigenous knowledge, local leadership and trust-based communication networks. The findings suggest that disaster communication policies should move beyond infrastructure-centred approaches and prioritise capacity building, recognition of local knowledge systems and participatory communication mechanisms. Such strategies can help ensure that EWSs function not only as technological tools but also as socially embedded communication processes that support efforts to reduce vulnerability and strengthen long-term community resilience in high-risk coastal environments.
